# The Action of Salicylic Acid upon Teeth

**Published:** 1885-05

**Authors:** Chas. Mayr

**Affiliations:** Springfield, Mass.


### THE ACTION OF SALICYLIC ACID UPON TEETH.

BY PROF. CHAS. MAYR, SPRINGFIELD, MASS.

To determine the exact action of salicylic acid upon teeth, on December 11, 1884, I put a freshly extracted tooth, in its natural condition of moisture and organic substance, weighing 1810 mgrs. (30.2 grains) into a solution, or rather mixture of 1230 mgrs. (25 grains) of salicylic acid, with 40 c. c. m. ($1\frac{1}{3}$ fl'd oz.) of water.

On December 26th, after having shaken up the mixture repeatedly, I took the tooth out. The enamel had not suffered very much, but the roots were softened to a considerable depth; from the surface of the enamel, which otherwise did not appear to be especially softened, I scraped off a powder weighing 5.7 mgrs. ($\frac{1}{10.5}$ grains), and containing 14 per cent. of organic substance. The tooth weighed 1720 mgrs. ($26\frac{2}{3}$ grains); from this it would appear that it had lost about 90 mgrs. of lime salts ($1\frac{3}{7}$ grains). But this assumption would be erroneous. The whole liquid, water, salicylic acid and dissolved lime salts, was evaporated and ignited, and it showed that 160 mgrs ($2\frac{5}{16}$ grains) of lime salts had been removed, and, as it appears from the above figures, partly substituted by water. I had first carefully analyzed the salicylic acid, and found it free from all inorganic substances. The inorganic salts which the salicylic acid had removed from the tooth had the following composition:

| | | | |
|---|---|---|---|
| Lime, (Ca. O.) | 30.2 | per cent. | |
| Magnesia, (Mg. O.) | 6.5 | " | |
| Phosphoric acid, ($P_2$ $O_5$) | 38.5 | " | with a trace of silica. |
| Carbonic acid, (C $O_2$) | 24.8 | " | |
| | 100 | | |

From these facts it is plain that, in 360 hours, a large excess of salicylic acid acting upon a tooth, removed 160 mgrs. ($2\frac{2}{3}$ grains) of lime salts. This gives the removal of about $\frac{1}{2}$ mgr. ($\frac{1}{120}$ grain) of lime salts in one hour, and the whole tooth would be decalcified in 150 days. If a preparation of salicylic acid is put into a tooth, the rate of decomposition of that tooth by the acid is probably much slower still. The tooth experimented on had a carious cavity. The chemical action of the salicylic acid upon the mass in the cavity was apparently the same as on the sound tooth. For a short application, therefore, the salicylic acid is not very dangerous, but like all small actions it will amount to a good deal in time. The tissue decalcified by salicylic acid is otherwise kept healthy, hence the damage is less than that from a decalcification by vinegar or sulphuric acid.

